# Demonstrative Midwifery

**Published:** 1851-10

**Authors:** 


We have been favored in the way described by our confrêre of the *Northern Lancet:*

*Demonstrative Midwifery.*—We have been favored with no less than *four* copies of the *Buffalo Courier*, of the 24th of June, and our attention forcibly attracted by imposing black lines encircling an article headed "Demonstrative Midwifery, again and for the last time." We have also received a copy of a Christian paper, published in the same city, and the name of which we did not deem it worth while to remember, reproducing the same article, and, therefore, plainly indicating that the same cowardly "NANCY" is the writer of both, and conceals herself under the obscurity of the editorial garb, to detract from Prof. White's motives, and to plume herself with the verdict of the American Medical Association.

In our number for November, 1850, we very plainly expressed our views in relation to Demonstrative Midwifery, we heartily commended the Professor's course, and in as free language expressed our contempt for the "XVII." We re-assert the same sentiments, and say to the Editor of the Courier and to his clerical brother, that our opinion is in nowise altered by the action of the Association on the subject. The Association, like every other body of men, whether legislative, scientific, or political, while they enact sound laws, give satisfactory deductions, or work for the "greatest good of the greatest number," are, nevertheless as truly guilty of many foolish and absurd things. The veto of the Association does not weigh one particle with us, and we know of very many others who will not in the least be affected by it.

We sincerely trust for the advancement of science, and the benefit of humanity, that this only true mode of imparting sound knowledge, of a most difficult and responsible department of our profession, will not be stayed by the clamors of ignorant, prejudiced, and cowardly men, in and out of the profession, but that Prof. White and his associates in the University of Buffalo will persevere in the course they have adopted, and which must reflect to their honor, and to the credit of their School.—*Northern Lancet.*

